# PReS-FINAL-2368: A, B, C, don't ever forget the joints

**DOI:** 10.1186/1546-0096-11-S2-O33

**Published:** 2013-12-05

**Authors:** W Abusrewil, A Whiteley, A -M McMahon, M Al-Obaidi

**Affiliations:** 1 Paediatric Department, Leeds General Infirmary, NHS, Leeds, UK; 2 Paediatric Rheumatology Department, Sheffield Children's Hospital, NHS, Sheffield, UK

## Introduction

The musculoskeletal examination has been shown to be poorly documented in the UK, despite musculoskeletal problems being common in children. Recent research has shown that examination of the joints is highly acceptable to patients and parents The musculoskeletal examination has been included in the Royal College of Pediatrics and Child Health clinical examination since 2009 and teaching resources are widely available. The pGALS examination has been validated and there are published red flags and triggers for the musculoskeletal examination.

## Objectives

To assess if the musculoskeletal (MSK) examination was being performed, in eight pediatric centers across Yorkshire, when the clinical situation would suggest it was warranted.

## Methods

397 patient's notes were randomly collected and reviewed retrospectively. Each centre assessed approximately 50 sets of patient's notes Patients were included if they had presented to hospital between August and November 2012. That single admission only was reviewed for this audit. The admission notes were reviewed for multiple parameters, including specifically looking for evidence of the triggers and red flags for the musculoskeletal examination. The notes were assessed to find out whether an MSK examination had been done by the doctors on the initial assessment and then the first and second reviews, and if there were triggers or red flags for the examination.

The notes were also assessed to see if there was evidence of documentation of the other clinical examinations (for example the cardiovascular system), as well as other variables, including the grade of the doctors reviewing patients. The information was collected on excel spreadsheets and then collated by the audit team to make some general conclusions about trends across Yorkshire.

## Results

35% of the 397 admissions had a trigger for an MSK examination. 26% of the 397 admissions had a red flag for an MSK examination.

Not a single patient who needed an MSK examination on initial assessment or first review had a full MSK examination documented, whereas 80% of patients routinely had a respiratory and cardiovascular examination on initial assessment. 22% had a partial musculoskeletal examination documented on initial examination. Only 1 out of 105 patients who had red flags for an MSK examination had a complete examination documented.

## Conclusion

In 2004 the musculoskeletal examination was shown to be poorly documented. This audit shows that the musculoskeletal examination is still rarely being documented or performed across Yorkshire, across disciplines, by consultants or more junior doctors, even when there are clear triggers and red flags for the examination.

We propose a change in the admission documentation regionally to include a specific MSK section and plan to re-audit following the introduction of this intervention. We also propose a national audit should be performed to create awareness nationally about these very disappointing results and if this picture is mirrored across the country then more educational measures, directed at all levels of doctors should be driven forward on a national level to ensure that this picture improves. We conclude that the musculoskeletal examination should be performed and documented routinely as part of the assessment of pediatric patients admitted to any discipline within the hospital.

## Disclosure of interest

None declared.

